# Deficiency of synaptotagmin-1 aggravates pressure overload-induced cardiac hypertrophy and dysfunction via the p38 MAPK signaling pathway in mice

**DOI:** 10.1007/s13577-025-01220-z

**Published:** 2025-04-25

**Authors:** Jing Shen, Junqiu Miao, Lifei Wu, Deping Wang, Guang Li, Haixiong Wang, Jimin Cao

**Affiliations:** 1 Key Laboratory of Cellular Physiology at Shanxi Medical University, Ministry of Education, and the Department of Physiology, Shanxi Medical University, Taiyuan, 030001 China; 2 Key Laboratory of Medical Electrophysiology at Southwest Medical University, Ministry of Education, and the Institute of Cardiovascular Research, Southwest Medical University, Luzhou, 646099 China; 3 Department of Cardiology, Shanxi Cardiovascular Hospital, Taiyuan, 030000 China

**Keywords:** Synaptotagmin-1, Cardiac hypertrophy, Cardiac fibrosis, Apoptosis, p38 MAPK

## Abstract

Cardiac hypertrophy is a major risk factor for heart failure and sudden cardiac death, but its molecular mechanisms have not been well clarified. Synaptotagmin-1 (SYT1) is an important regulator of exocytosis and apoptosis and has been found expressed in the myocardium, while its functions in heart diseases have rarely been studied. Here, we investigated the role and mechanism of SYT1 in pressure overload-induced cardiac hypertrophy. Transverse aortic constriction (TAC) surgeries were performed to induce cardiac hypertrophy in global *Syt1* knockout (*Syt1*^+/−^) mice and C57BL/6J wild-type (WT) littermates in vivo, with respective sham mice as negative controls. Cardiomyocyte hypertrophy was induced by angiotensin II (Ang II) in H9C2 cells in vitro. The results showed that SYT1 expression was significantly upregulated in WT-TAC mice and in Ang II-treated H9C2 cells. Blocking angiotensin receptor by losartan decreased SYT1 expression in Ang II-treated H9C2 cells. *Syt1*^+/−^ mice showed significantly exacerbated cardiac hypertrophy, dysfunction, fibrosis, apoptosis and phosphorylation of myocardial p38 MAPK in response to TAC compared to WT mice. Knocking down SYT1 using siRNA in H9C2 cells aggravated Ang II-induced cell hypertrophy and apoptosis, and also enhanced p38 MAPK phosphorylation. Inhibition of p38 MAPK by SB203580 significantly alleviated the hypertrophy and apoptosis in Ang II-treated H9C2 cells. We conclude that deficiency of SYT1 aggravates pressure overload-induced cardiac hypertrophy via the p38 MAPK signaling pathway. The study elucidates a novel role of SYT1 in cardiac remodeling.

**Supplementary Information:**

The online version contains supplementary material available at 10.1007/s13577-025-01220-z.

## Introduction

Cardiovascular disease is still the leading cause of death at a global scale. Cardiac hypertrophy induced by variant continuous hypertrophic stimuli, such as pressure overload, neurohumoral activation and genetic alterations, is the common stage of many cardiovascular disorders [1, 2]. Cardiac hypertrophy is characterized by cardiomyocyte hypertrophy and apoptosis, and myocardial fibrosis. These changes collectively contribute to cardiac remolding and dysfunction. Without intervention, the hypertrophic heart will further deteriorate, leading to serious consequences such as heart failure and even sudden cardiac death. Although substantial achievements have been made in studying the mechanisms, there are rare effective treatments for myocardial hypertrophy. Therefore, further explorations on the mechanisms and effective treatment targets of cardiac hypertrophy are still necessary and urgent.

Synaptotagmins (SYTs) are a family of membrane-trafficking proteins, and have been well documented in cellular vesicle exocytosis [3]. The biological functions of SYTs beyond vesicle release, however, tend to be less characterized and underestimated. Seventeen synaptotagmin isoforms (SYT1 − SYT17) have been identified to date with differences in their molecular structures, tissue distributions, and cellular functions [4]. Of these, SYT1 is the most abundantly studied. In addition to its typical function in cell exocytosis, SYT1 plays a complex role in the regulation of cell activities including apoptosis. For example, SYT1 weakens neuronal apoptosis [5]. SYT1 overexpression particularly decreases the neuronal cell apoptotic rate in a mouse model of Parkinson’s disease, which subsequently exerts a neuroprotective effect [6]. In addition, SYT1 restoration affects cell apoptosis in colon carcinoma [7], thyroid cancer [8], and glioblastoma [9]. Thus, SYT1 is considered an important regulator of apoptosis in nervous system and tumors. However, the role of SYT1 in cardiomyocyte hypertrophy and apoptosis has not been elucidated. Importantly, cardiomyocyte apoptosis plays an important role in the transition of cardiac hypertrophy to heart failure, and apoptosis may be an important intervention target to improve myocardial hypertrophy. From this point, exploring the role of SYT1 in cardiomyocyte apoptosis may clarify its role in myocardial hypertrophy.

The mitogen-activated protein kinase (MAPK) pathway is closely related to cardiac hypertrophy. MAPKs regulate multiple physiological processes, including cellular homeostasis, cell proliferation, apoptosis and stress responses, which are highly active in hypertrophic hearts. Protein 38 mitogen-activated protein kinase (p38 MAPK), a critical subfamily member of MAPKs, can be phosphorylated and activated by a variety of stimuli, such as pressure overload, ischemia and hypoxia. Once activated, p38 MAPK contributes to cardiomyocytes apoptosis, leading to cardiac hypertrophy, dysfunction, and failure. It has been confirmed that inhibiting the p38 MAPK signaling pathway can alleviate myocardial apoptosis and cardiac hypertrophy. A study conducted by Shi et al. revealed that overexpression of SYT1 inhibits the MAPK signaling pathway and MAPK phosphorylation in colorectal cancer cells [10]. It is possible that SYT1 deficiency may exacerbate cardiomyocyte apoptosis by activating the p38 MAPK pathway.

The present study aimed to investigate the expression and function of SYT1 in the development of cardiac hypertrophy both in vivo and in vitro. We found that the SYT1 expression levels were significantly upregulated in both the mouse model of TAC-induced cardiac hypertrophy and in the H9C2 cardiomyocyte model of Ang II-induced hypertrophy. Knocking out SYT1 in vivo or silencing SYT1 in vitro respectively exacerbated TAC- or Ang II-induced cardiac injury including cardiac hypertrophy, fibrosis, dysfunction and cardiomyocyte hypertrophy and apoptosis, suggesting a protective role of SYT1 in cardiac hypertrophy. The cardioprotective effect of SYT1 was associated with suppression of p38 MAPK phosphorylation. The study suggests that targeting SYT1 may have clinical perspectives in the treatment of cardiac hypertrophy. To our knowledge, this is the first study to demonstrate the protective role of SYT1 in cardiac hypertrophy.

## Materials and methods

### Experimental animals

*Syt1* heterozygous knockout (*Syt1*^+*/−*^) mice were constructed on the C57BL/6J background by deleting the exon 6 to exon 8 in *Syt1* gene using the CRISPR/Cas9 approach. *Syt1*^*−/−*^ mice were not used as they usually died after birth. WT littermates were used as controls. Genotype identification of *Syt1*^+*/−*^ mice was performed based on PCR assay using genomic DNA extracted from mouse tails. *Syt1*^+*/−*^ mice and WT littermates were housed in cages (4 − 5 mice per cage) under a 12:12 h light: dark cycle and had free access to regular chaw and water. Male mice of all the strains were used in the experiments. Experimental procedures involving the care and use of animals were approved by the Animal Care and Use Committee of Shanxi Medical University (Approval ID-SYDL2024007) and the experimental protocols conformed to the Guide for the Care and Use of Laboratory Animals published by the U.S. National Institutes of Health.

### Transverse aortic constriction (TAC)

TAC was performed to induce cardiac hypertrophy in 8–10 weeks old male mice. Mice of both strains (*Syt1*^+*/−*^ and WT) were anesthetized with 2% isoflurane inhalation. Animal were then limb fixed in a supine position. A median thoracotomy was performed at the level of the first intercostal space. The aortic arch between the innominate and left common carotid arteries was isolated and transversely constricted using a 27 G cannula. The needle was immediately removed, leaving a stenotic lumen in the aortic arch. Sham mice underwent an identical surgical procedure without aortic banding. Finally, the chest walls were closed and animals were allowed to live for 8 weeks after TAC.

### Echocardiography

Doppler echocardiography for small animals were performed to measure cardiac structure and function of the mice eight weeks after TAC or sham surgery. A Prospect 3.0 high-resolution in vivo imaging system (S-Sharp) with a scanhead (a 30–50 MHz transducer) was used for echocardiographic analysis. Mice were anesthetized with 2% isoflurane inhalation before analysis. M-mode images and two-dimensional images were obtained in a short-axis view. Echocardiographic parameters including LV diastolic and systolic internal dimensions (LVIDd, LVIDs), LV diastolic and systolic posterior wall thickness (LVPWd, LVPWs) and left ventricular ejection fraction (LVEF) and left ventricular fractional shortening (LVFS) were then measured and analyzed.

### Histology

Eight weeks after TAC or sham surgery, mice were deeply anaesthetized with isoflurane, hearts were transversely perfused via the aortae by ice-cold 1 × PBS, and then hearts were harvested. Heart tissues were fixed with 10% neutral buffered formalin for overnight, then embedded in paraffin, and cut to 5-μm sections. Tissue sections were deparaffinized and then stained with hematoxylin & eosin (H&E) or Sirius red to evaluate the microstructures including hypertrophy and fibrosis. To perform H&E staining, the sections were soaked with hematoxylin for 3 min and then washed with running tap water for 1 min. Then, the sections were blued with 0.1% ammonia water, followed by staining with 0.1% eosin for 2 min. The sections were dehydrated with ethanol and transparentized with xylene, and sealed with neutral resin. To perform Sirius red staining, tissue sections were stained with Sirius Red staining kit (G1472, Solarbio Life Science, Beijing China). The sections were incubated in 0.1% Sirius red solution for approximately 8 min and then rinsed with absolute alcohol, followed by xylene transparency and sealed with neutral gum. The stained sections were visualized and photographed under a microscope (Olympus, Tokyo, Japan) at a 20 × magnification. For fibrosis area quantification, at least ten random fields of each sample were analyzed. Briefly, the stained-red fibrotic areas were measured by adjusting the color-based threshold. The total fibrosis areas and the percentage of fibrosis area over the whole inspected areas in each slide were calculated [11] using the Image J software (National Institutes of Health).

### RNA extraction and quantitative real-time PCR

The total RNA was isolated from the left ventricular (LV) tissues using Trizol reagent according to the manufacturer’s protocol. Extracted RNA was reversely transcribed to cDNA using PrimeScript RT reagent kit (RR064A, TaKaRa, Toyobo, Japan). Real-time quantitative PCR (qPCR) was performed by using SYBR premix Ex Taq (RR420, TaKaRa, Toyobo, Japan) on a QuantStudio real-time PCR system (Life Technologies, Thermo Fisher Scientific). PCR primer sequences used were listed below.

ANP: forward: 5′-TCTTCCTCGTCTTGGCCTTT-3′
  reverse: 5′-CCAGGTGGTCTAGCAGGTTC-3′
BNP: forward: 5′-TGGGAGGTCACTCCTATCCT-3′
  reverse: 5′-GGCCATTTCCTCCGACTTT-3′
β-MHC: forward: 5′-ACTGTCAACACTAAGAGGGTCA-3′
  reverse: 5′-TTGGATGATTTGATCTTCCAGGG-3′
Col1a1: forward: 5′-CTGGCGGTTCAGGTCCAAT-3′
  reverse: 5′-TTCCAGGCAATCCACGAGC-3′
Col3a1: forward: 5′-TGAATGGTGGTTTTCAGTTCAG-3′
  reverse: 5′-GATCCCATCAGCTTCAGAGACT-3′
GAPDH: forward: 5′-AGAACATCATCCCTGCATCC -3′
  reverse: 5′-AGTTGCTGTTGAAGTCGC-3′

### Western blotting

Small pieces of mouse left ventricular tissues or H9C2 cells were homogenized in RIPA buffer (Beyotime, Shanghai, China) to extract total proteins. The protein concentrations in samples were measured using the BCA protein assay kit (Beyotime, Shanghai, China). Extracted proteins were separated with 10% SDS-PAGE and then were transferred onto PVDF membranes (Millipore, Billerica, MA, USA), followed by treatment with the blocking buffer. The membranes were incubated with respective primary antibodies at 4 °C for overnight. After four 5-min washes in TBST, the membranes were incubated with HRP-conjugated secondary antibody for 1 h. Then the positive blots in the membranes were visualized by autoradiography using ChemiDoc™ (Bio-Rad Laboratories, Hercules, CA, USA). β-actin was used as an internal control. The antibodies used were: anti-SYT1 (1:1000, ab254104, Abcam, Cambridge, UK), anti-B cell lymphoma-2 (Bcl-2) (1:1000, ab196495, Abcam, Cambridge, UK), anti-Bcl 2-associated X (Bax) (1:1000, ab32503, Abcam, Cambridge, UK), anti-p-p38 (Thr180/Tyr182) (D3F9) (1:1000, #4511, CST, Danvers, MA,USA), anti-p38 (D13E1) (1:1000, #8690, CST, Danvers, MA, USA), anti-p-JNK (Thr183/Tyr185) (81E11) (1:1000, #4668, CST, Danvers, MA, USA), anti-JNK (1:1000, #9252, CST, Danvers, MA, USA), anti-p-ERK1/2 (Thr202/Tyr204) (D13.14.4E) (1:1000, #4370, CST, Danvers, MA, USA), anti-ERK1/2 (137F5) (1:1000, #4695, CST, Danvers, MA, USA), and anti-β-actin (13E5) (1:1000, #4970, CST, Danvers, MA, USA). The secondary antibodies used were goat antimouse (1:1000, ZB-2305, Zhongshan Company, Beijing, China) and goat antirabbit (1:1000, ZB-2301, Zhongshan Company, Beijing, China).

### Enzyme-linked immunosorbent assay (ELISA) analysis

Ang II levels in left ventricular tissues and serum were measured using Ang II ELISA kits (JL31138-96 T, JONLNBIO, Shanghai China) following the manufacturer’s instructions. Briefly, the microplate wells are precoated with mouse Ang II. Then the Ang II standards, samples and biotin-labeled antibody were added into the microplate and incubated at 37 °C for 1 h. After washing, 100 µL of HRP conjugated streptavidin was added to the microplate and incubated for 30 min and washing, followed by adding 100 µL of 3,3´,5,5´-tétraméthylbenzidine (TMB) for color development. The reaction was stopped with 50 µL/well of stopping solution, and the absorbance was measured at 450 nm. By applying a four-parameter fit to the standard curve, the concentrations of Ang-II in the samples were determined.

### TUNEL staining

Myocardial apoptosis was examined using TUNEL reaction kit (Boster, Wuhan, China). Paraffin sections were incubated with protease K solution at 37 °C for 20 min and washed with TBS for 2 min. The sections were then stained with TdT labeling solution at 37 °C for 2 h, rinsed with TBS for three times, incubated with blocking buffer for 30 min and incubated with anti-DIG-biotin at 37 °C for 60 min, washed with TBS for 5 min, stained with DAPI solution for 5 min at room temperature. Positive TUNEL signals were observed and photographed under a fluorescence microscope.

### Cell culture and treatment

The embryonic rat heart-derived H9C2 cells were purchased from SEVEN BIOTECH (AC601, Beijing, China). Cells were cultured in high glucose Dulbecco’s modified Eagle’s medium (H-DMEM) (SEVEN, Beijing, China) supplemented with 10% fetal bovine serum (FBS) (Hyclone, USA), 100 units/ml penicillin and 100 μg/ml streptomycin at 37 °C and 5% CO_2_. Cells were stimulated with 1 μM Ang II (05–23-0101, Sigma, USA) to induce hypertrophy. Cells treated with equivalent saline were used as a control. To inhibit p-38 MAPK, cells were pretreated with 1 μM SB203580 (S8307, Sigma, USA) for 1 h. To knock down *Syt1* expression in H9C2 cells, siRNA (Anbote, Jinan, China) and the negative control siRNA were mixed with Lipofectamine 2000 (Thermo Fisher Scientific, USA) for transfection according to the manufacturer’s instruction. Cells were then harvested 24 h after transfection for further analysis.

### Immunofluorescent staining

Paraffin sections were incubated with 0.1% Triton X-100 for 20 min and then rinsed with PBS for 15 min. Following incubation in 5% goat serum at 37 °C for 1 h, sections were incubated overnight with primary antibodies against α-SMA (1:500, ab179467, Abcam, Cambridge, USA) at 4 °C. The sections were washed and then incubated with fluorescence-labeled secondary antibody (1:500, P11047, Thermo Scientific, Waltham, MA, USA) for 1 h at room temperature. Afterwards, sections were counterstained with DAPI solution for 2 min at room temperature in a humidifying box and photographed under a luminescence microscope.

### Statistical analysis

The results were presented as mean ± standard error (SEM). The data were analyzed and graphed using GraphPad Prism 7.0 (GraphPad Software, In., San Diego, CA, USA). Statistical differences were analyzed by grouped *t* test for two groups or one-way ANOVA followed by Turkey’s multiple comparison for three or more groups. The Kaplan–Meier method with log-rank test was used for survival analysis. *P* < 0.05 were considered statistically significant.

## Results

### Differential phenotypes of Syt1^−/−^ and Syt1^+/−^ mice

**Fig. 1:**
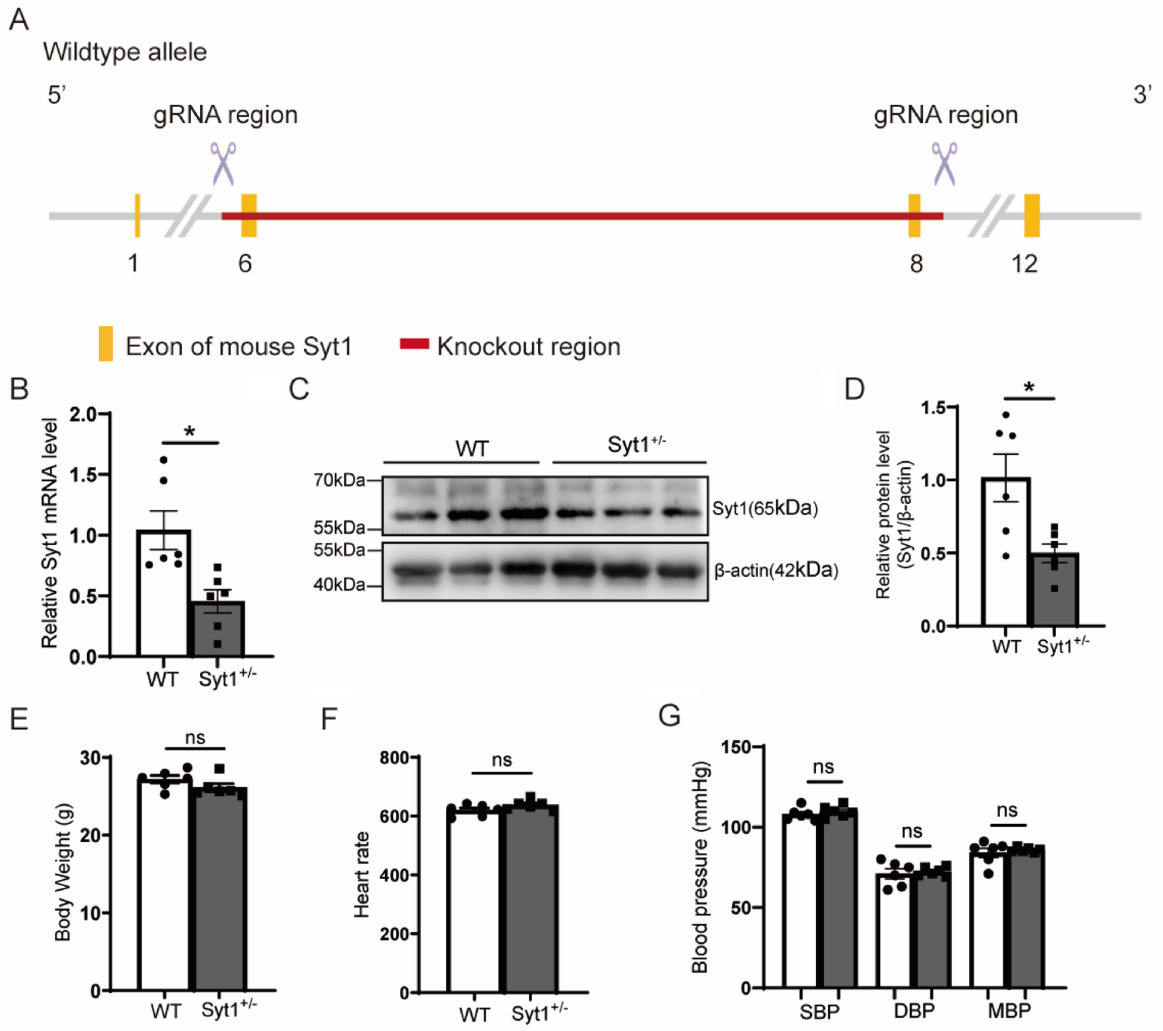
Generation and identification of SYT1 global knockout mice. **A** schematic overview depicting the targeting strategy for knocking out *Syt1*. **B** cardiac mRNA levels of *Syt1* in hearts of WT mice and *Syt1*^+*/−*^ mice (*n* = 6). **C** and **D** Western blots and respective quantitative results of SYT1 levels in the hearts taken from WT mice and *Syt1*^+*/−*^ mice. β-actin was used as loading control (*n* = 6). **E** body weight. **F** heart rate. **G** blood pressure of *Syt1*^+*/−*^ mice and WT littermates (*n* = 6 for each strain). Mean ± SEM. *P < 0.05. *ns* not statistically significant

To obtain more insight into the role of SYT1 in cardiac hypertrophy, *Syt1* knockout (KO) mice were generated and identified (Fig. 1A). Male or female *Syt1* homozygous KO (*Syt1*^*−/−*^) mice were born dead, as also reported in a previous study [12]. Heterozygous *Syt1* KO (*Syt1*^+*/−*^) mice showed no abnormality either in physical condition or in behavior, and could mate and generate offspring, therefore were used in the present study. We selected male mice to perform the experiments. The mRNA and protein expression levels of cardiac SYT1 were detected using RT-qPCR and western blotting. The results showed that both the mRNA (Fig. 1B) and protein (Fig. 1C, D) expression levels of SYT1 were markedly decreased in the hearts of *Syt1*^+*/−*^ mice when compared with the WT mice. No statistical differences were found in the body weight (Fig. 1E), heart rate (Fig. 1F) and blood pressure (Fig. 1G) between the 16-week-old *Syt1*^+*/−*^ mice and the WT littermates. These results revealed that *Syt1*^+*/−*^ mice were successfully generated and could be used in the following experiments.

### Upregulation of SYT1 expression in the hearts of TAC mice and in Ang II-treated H9C2 cells

**Fig. 2:**
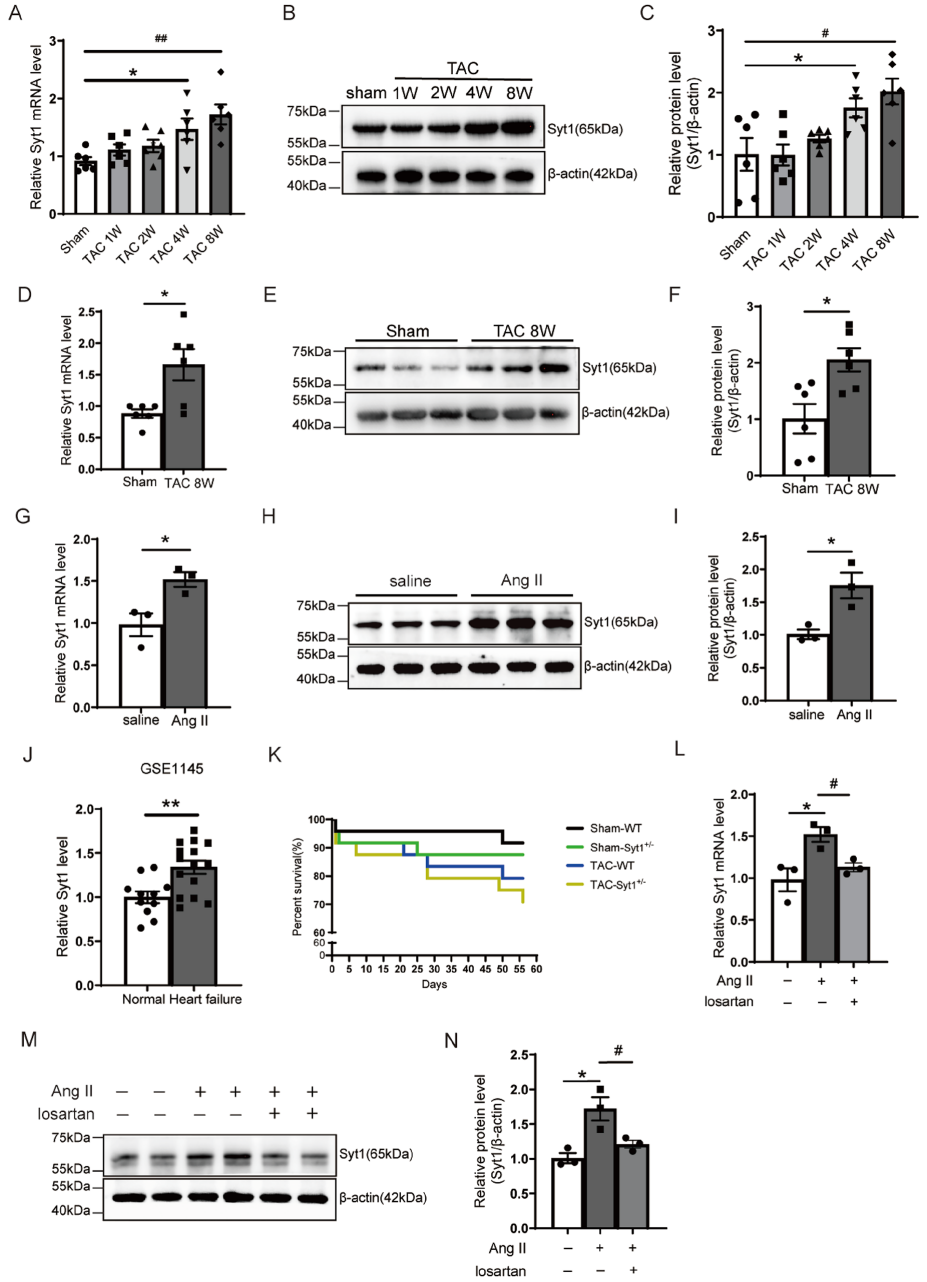
Upregulation of SYT1 in hypertrophic hearts. **A** mRNA levels of SYT1 in the hearts of WT mice 1, 2, 4, and 8 weeks after TAC (*n* = 6). **B** and **C** Western blots and respective quantitative results of SYT1 levels in the hearts of WT mice 1, 2, 4, and 8 weeks after TAC (*n* = 6). β-actin was used as loading control. **D** mRNA levels of SYT1 in the hearts of WT 8 weeks after TAC (*n* = 6). **E** and **F** Western blots and respective quantitative results of SYT1 levels in the hearts of WT mice 8 weeks after TAC (*n* = 6). β-actin was used as loading control. **G** relative mRNA levels of SYT1 in H9C2 cells treated with Ang II for 24 h (*n* = 3).** H** and **I** Western blots and respective quantitative results of SYT1 levels in H9C2 cells treated with Ang II for 24 h (*n* = 3). **J** relative mRNA levels of SYT1 in human cardiac tissues derived from GSE1145 dataset (*n* = 11 for normal hearts, *n* = 15 for failing heart). **K** survival curves of mice 8 weeks after TAC (*n* = 24 per group). **L** relative SYT1mRNA levels in H9C2 cells treated with Ang II or Ang II + losartan for 24 h (*n* = 3). **M** and **N** Western blots and respective quantitative results of SYT1 protein levels in H9C2 cells treated with Ang II or Ang II + losartan for 24 h (*n* = 3). Mean ± SEM. *P < 0.05. **P < 0.01. ^#^*P* < 0.05. ^##^*P* < 0.01

To explore whether SYT1 was pathologically relevant to cardiac hypertrophy, we executed the animals and examined the mRNA and protein expression levels of SYT1 in the LV tissues of mice respectively at 1, 2, 4, and 8 weeks after TAC. SYT1 expression was upregulated after TAC over time and reached to peak on week 8 compared with the sham mice (Fig. 2A–C). Therefore, 8 weeks after TAC was selected as the end point of cardiac hypertrophy development in this study. Next, we subjected mice to TAC surgery for 8 weeks to verify the expression of SYT1 during pressure overload. The results showed that SYT1 mRNA level was significantly elevated in the LV of WT-TAC mice when compared with the WT-sham mice (Fig. 2D). The SYT1 protein level was also elevated in the LV of WT-TAC mice compared with the WT-sham mice (Fig. 2E, F). Similarly, the mRNA and protein levels of SYT1 in Ang II-stimulated H9C2 cardiomyocytes were also notably elevated compared to the H9C2 cells treated with saline (Fig. 2G − I). These results suggest that SYT1 is involved in pressure overload-induced cardiac hypertrophy. We also analyzed the SYT1 expression in human hearts by mining the GEO dataset GSE1145 which contained 26 human heart samples (normal hearts, *n* = 11; failing hearts, *n* = 15). Similar to our animal study, GSE1145 analysis showed that the SYT1 mRNA level was elevated in human failing hearts as compared to that of normal hearts (Fig. 2J). The survival curves of mice showed that there was a rapid and high rate of death among mice after TAC, and *Syt1*^+/−^ mice yielded a higher mortality than their WT littermates 8 weeks after TAC (Fig. 2K), but it did not reach statistical significance (*P* = 0.5284). The effect of angiotensin receptor blockade on SYT1 expression was checked by using losartan (HY-17512, MCE, New Jersey, USA) in H9C2 cells. The results show that the mRNA and protein levels of SYT1 were notably elevated in Ang II-stimulated H9C2 cells as compared to the H9C2 cells treated with saline; blocking AT1 receptor by losartan significantly decreased the mRNA and protein expression levels of SYT1 in H9C2 cells as compared to H9C2 cells treated with Ang II alone (Fig. 2L–N). These results demonstrate that Ang II enhances SYT1 expression via AT1 receptor in cardiomyocytes.

### Aggravation of cardiac hypertrophy and dysfunction in Syt1^+/−^ mice after TAC stress

**Fig. 3:**
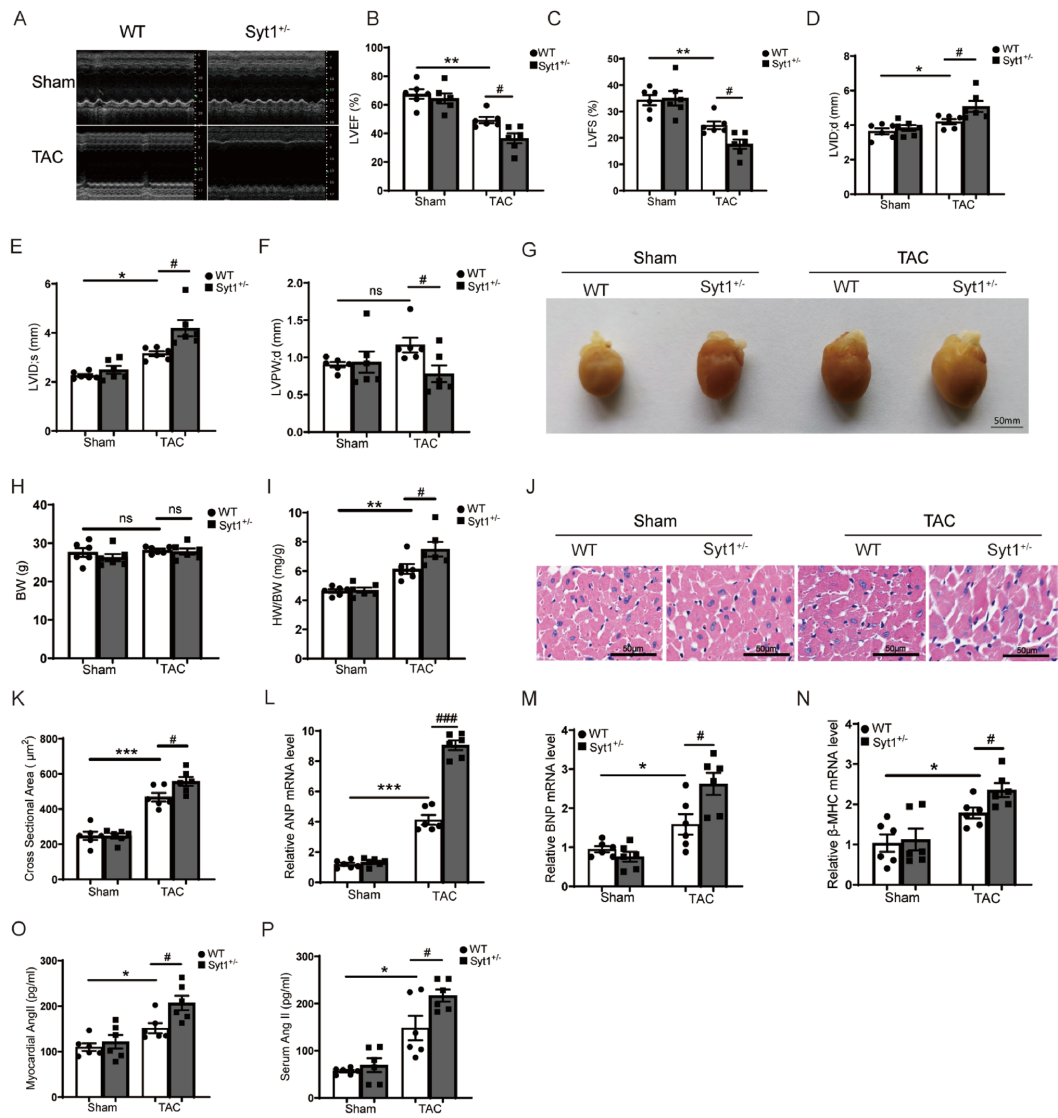
Aggravation of TAC-induced cardiac hypertrophy and dysfunction in *Syt1*^+*/−*^ mice. **A** representative M-mode echocardiographic images of left ventricular chamber (*n* = 6). **B** percent changes of LVEF. **C** percent changes of LVFS. **D** LVIDd. **E** LVIDs. **F** LVPWd derived from echocardiographic results (*n* = 6). **G** gross pictures of the hearts. **H** body weight of WT and *Syt1*^+*/−*^ mice 8 weeks after TAC or sham surgery (*n* = 6). **I** heart weight to body weight ratio (HW/BW) (*n* = 6). **J** and **K** representative images of H&E stains of LV tissue sections and semi-quantitative analysis of the relative cross-sectional area of cardiomyocytes (*n* = 6). **L** through **N** RT-qPCR results of myocardial ANP, BNP and β-MHC mRNA levels, respectively (*n* = 6). **O** and **P** myocardial and serum levels of Ang II in mice. Mean ± SEM. *P < 0.05, **P < 0.01, ***P < 0.001. ^#^*P* < 0.05, ^##^*P* < 0.01. *ns* not statistically significant

The typical M-mode echocardiographic images of the short-axis view were shown in Fig. 3A. Eight weeks after TAC, WT mice exhibited significant decreases in LVEF and LVFS, accompanied by increased thickness of LVIDd and LVIDs. WT-TAC mice showed an increased trend in their LVPWd, but it did not reach a statistical significance compared to the LVPWd of WT-sham mice. The LVEF, LVFS and LVPWd were significantly decreased, while the LVIDd and LVIDs were significantly increased, in *Syt1*^+*/−*^-TAC mice as compared to WT-TAC mice, indicating a severe cardiac hypertrophy and dysfunction in the *Syt1*^+*/−*^-TAC mice relatively to the WT-TAC mice (Fig. 3B − F). Representative gross images of hearts taken from *Syt1*^+*/−*^ and WT mice 8 weeks after TAC or sham surgeries were shown in Fig. 3G. The body weights of mice did not show significant differences among groups (Fig. 3H), while the heart weight to body weight ratio (HW/BW) were higher in *Syt1*^+*/−*^ mice than in WT mice 8 weeks after TAC (Fig. 3I), indicating that SYT1 deficiency aggravates TAC-induced cardiac hypertrophy. H&E stains showed that there was no significant difference in the cardiomyocyte cross-sectional area between *Syt1*^+*/−*^-sham mice and WT-sham mice, while the cross-sectional areas of cardiomyocytes in *Syt1*^+*/−*^-TAC mice were larger than the WT-TAC mice (Fig. 3J, K), suggesting cardiomyocyte hypertrophy. Furthermore, eight weeks after TAC, the myocardial expressions of classic cardiac hypertrophic factors, including atrial natriuretic peptide (ANP), brain natriuretic peptide (BNP) and myosin heavy chain β (β-MHC), were significantly upregulated in the hearts of *Syt1*^+*/−*^ mice compared to WT mice (Fig. 3L − N). We also investigated the involvement of angiotensin signaling in TAC-induced cardiac hypertrophy using ELISA. The results showed that TAC significantly increased the myocardial and serum Ang II levels of WT-TAC mice as compared to the WT-sham mice. In addition, the myocardial and serum Ang II levels were significantly higher in the *Syt1*^+/−^-TAC mice than in the WT-TAC mice (Fig. 3O, P). These results indicate that the deficiency of SYT1 aggravates TAC-induced cardiac hypertrophy and contractile dysfunction, and angiotensin signaling is involved in these events.

### Aggravation of cardiac fibrosis and apoptosis in Syt1^+/−^ mice after TAC stress

**Fig. 4:**
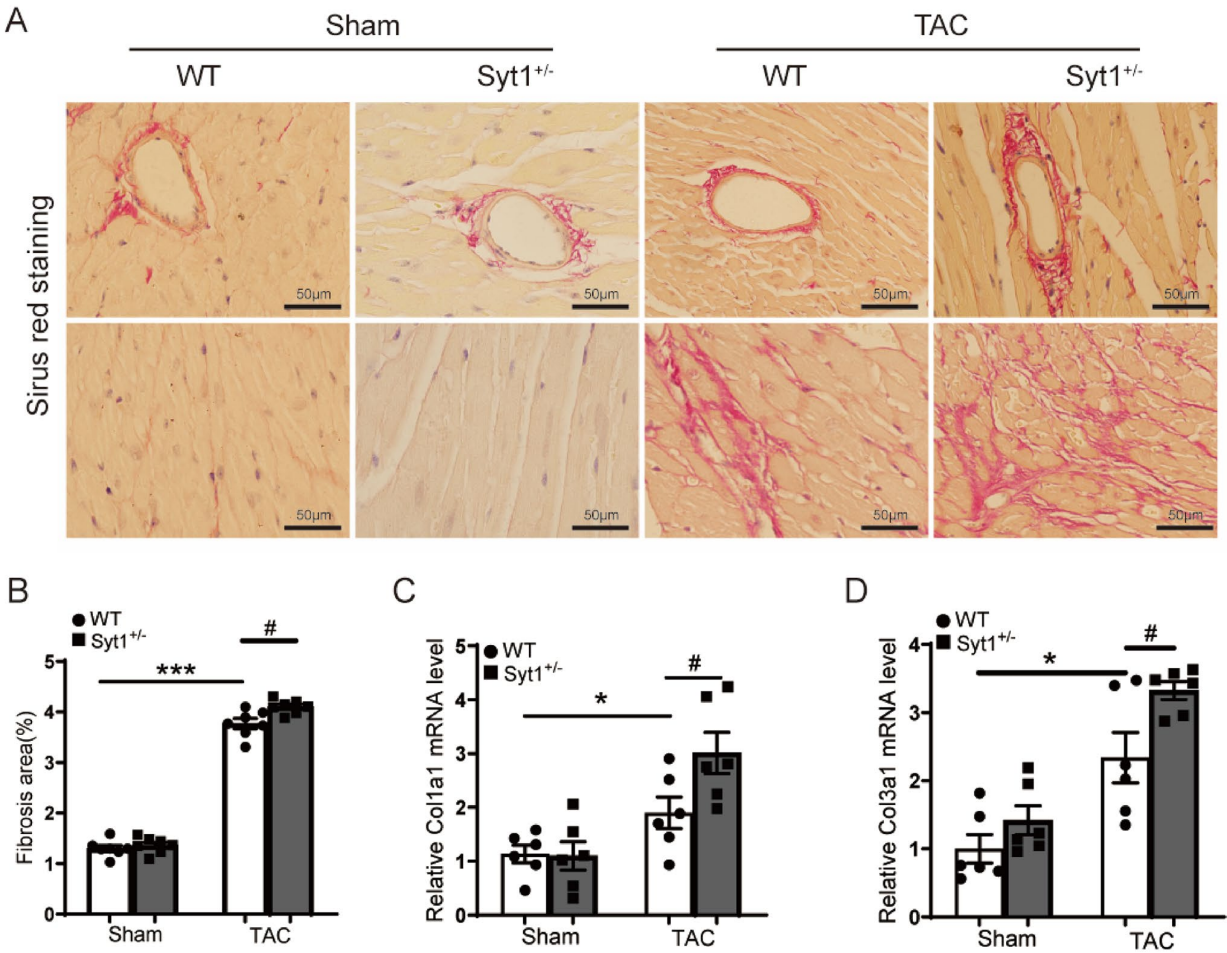
Exacerbation of TAC-induced cardiac fibrosis in *Syt1*^+*/−*^ mice. **A** representative Sirius red staining images of LV tissue sections (*n* = 6). **B** semi-quantification of relative fibrosis area (*n* = 6). **C** and** D** RT-qPCR results of *Col1a1* and *Col3a1* mRNA levels in the LV tissues of mice (*n* = 6). Mean ± SEM. *P < 0.05, ***P < 0.001. ^#^*P* < 0.05

Fibrosis is a feature of cardiac hypertrophy and remodeling. Sirius red stains showed increased fibrosis in the perivascular and interstitial areas of myocardium in the *Syt1*^+*/−*^-TAC mice compared to the WT-TAC mice (Fig. 4A, B). Meanwhile, the RT-qPCR analysis showed that TAC stress increased the mRNA levels of myocardial collagen I (Col1a1) and collagen III (Col3a1), the markers of fibrosis, in WT-TAC mice when compared with the WT-sham mice, while no differences were observed between *Syt1*^+*/−*^-sham mice and WT-sham mice (Fig. 4C, D). In addition, the mRNA levels of Col1a1 and Col3a1 were significantly increased in the cardiac tissues of *Syt1*^+*/−*^-TAC mice as compared to the WT-TAC mice (Fig. 4C, D), indicating cardiac fibrosis.

**Fig. 5:**
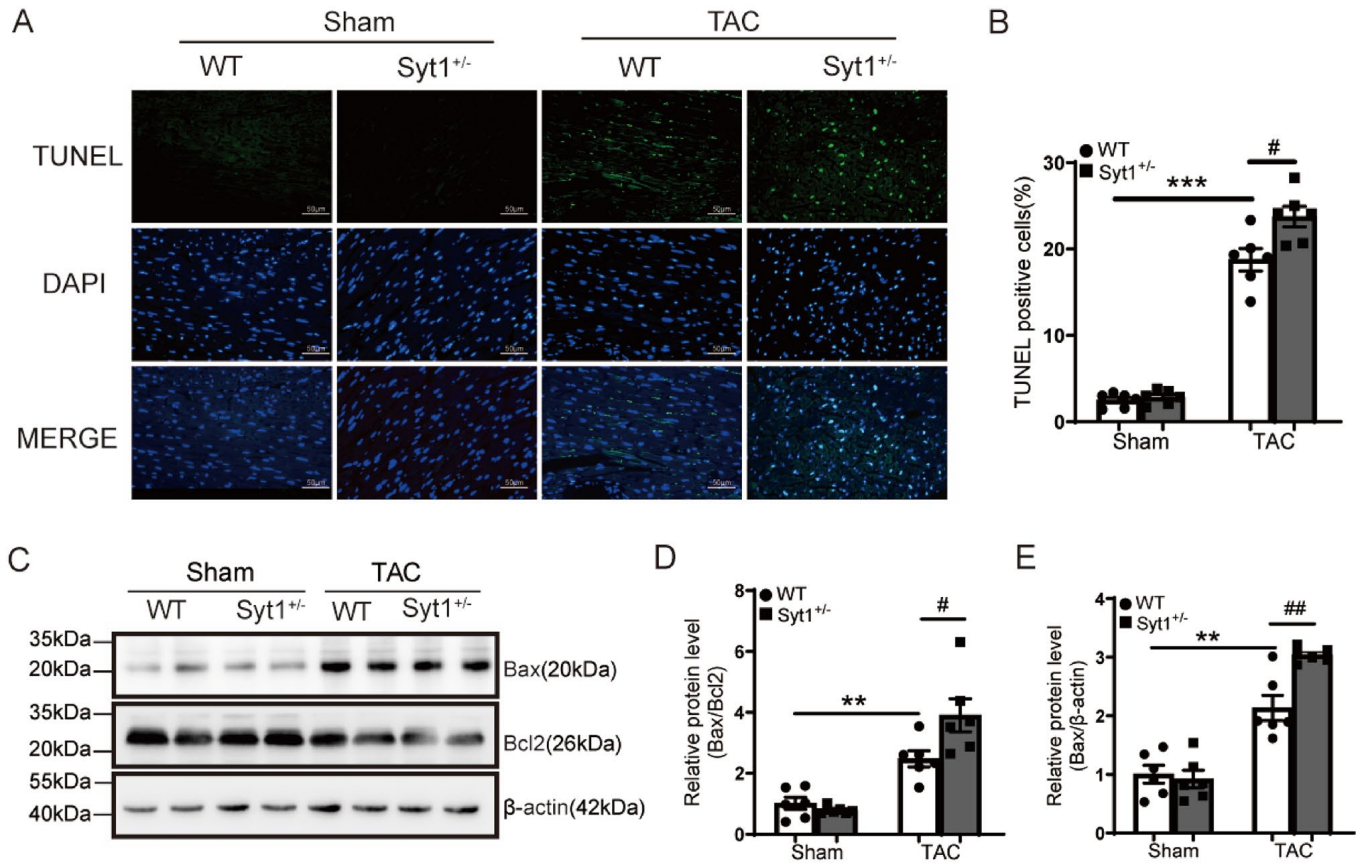
Aggravation of TAC-induced cardiac apoptosis in *Syt1*^+*/−*^ mice.** A** representative TUNEL staining images of mice LV tissue sections (*n* = 6). **B** quantification of the TUNEL-positive cells (*n* = 6). **C–E** Western blots and respective quantitative results of Bax and Bcl2 levels in the LV tissues from WT-sham, WT-TAC, *Syt1*^+*/−*^-sham and *Syt1*^+*/−*^-TAC mice (*n* = 6). Mean ± SEM. **P < 0.01, ***P < 0.001. ^#^*P* < 0.05, ^#^*P* < 0.01

Because apoptosis is an important character of cardiac pathological remodeling, and SYT1 contributes to apoptosis, we therefore examined the potential role of SYT1 in cardiomyocyte apoptosis in cardiac hypertrophy. A large number of TUNEL-positive cardiomyocytes were observed in the myocardium of WT-TAC mice as compared to the WT-sham mice (Fig. 5A, B). Notably, cardiomyocyte apoptosis (TUNEL-positive) was significantly exacerbated in *Syt1*^+*/−*^-TAC mice compared to the WT-TAC mice (Fig. 5A, B). In addition, western blots showed that the *Syt1*^+*/−*^-TAC mice exhibited higher myocardial Bax protein level and higher Bax/Bcl2 ratio than the WT-TAC mice (Fig. 5C − E), suggesting that SYT1 deficiency aggravates TAC-induced cardiomyocyte apoptosis.

### Exacerbation of hypertrophy and apoptosis by SYT1 silencing in H9C2 cardiomyocytes in response to Ang II challenge

**Fig. 6:**
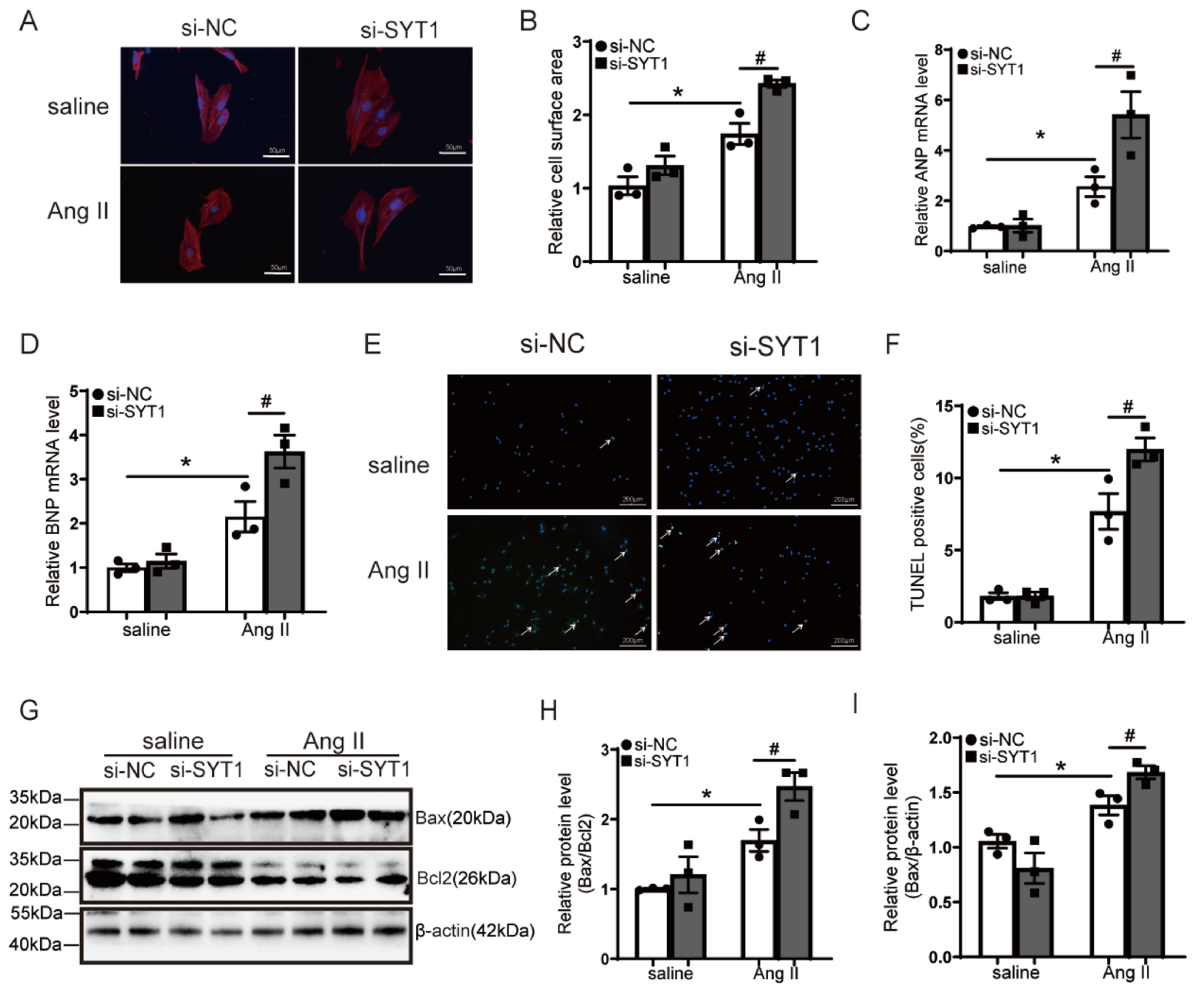
Enhancement of Ang II-induced H9C2 cell hypertrophy and apoptosis after SYT1 silencing. **A** representative immunofluorescent images of H9C2 cells infected with indicated siRNAs and treated with saline or Ang II for 24 h (*n* = 3). **B** quantitative measurements of H9C2 cell surface area reflecting cell size (*n* = 3). **C** and** D** RT-qPCR results of ANP and BNP levels in the H9C2 cells (*n* = 3). **E** representative TUNEL staining images of H9C2 cells (*n* = 3). **F** quantification of TUNEL-positive cells (*n* = 3). **G** through **I** Western blots and respective quantitative results of Bax and Bcl2 levels in H9C2 cells infected with the indicated siRNAs and treated with saline or Ang II for 24 h (*n* = 3). Mean ± SEM. *P < 0.05. ^#^*P* < 0.05

The above mice experiments demonstrated that deficiency of SYT1 exacerbated the progression of TAC-induced cardiac hypertrophy. We speculated this might due to the direct effect of SYT1 on cardiomyocytes. We addressed this hypothesis in H9C2 cardiomyocytes using the SYT1 siRNA approach. Results showed that silencing *Syt1* expression (si-SYT1) in H9C2 cardiomyocytes significantly enlarged the surface area of H9C2 cells compared with the negative control (si-NC) after Ang II stress (Fig. 6A, B), suggesting that SYT1 can protect cardiomyocytes from Ang II-induced hypertrophy. Accordingly, silencing *Syt1* expression in H9C2 cardiomyocytes further increased the transcription levels of ANP and BNP in H9C2 cells in response to Ang II challenge (Fig. 6C, D). These results indicate that SYT deficiency can directly enhance Ang II-induced cardiomyocyte hypertrophy.

We also investigated the effect of SYT1 on cardiomyocyte apoptosis. The proportion of TUNEL-positive cells (indicating apoptosis) in the Ang II group was much higher than that in the control group, and *Syt1* silencing further enhanced the apoptosis (Fig. 6E, F). Moreover, silencing *Syt1* expression increased the expression of Bax and decreased the expression of Bcl-2 in Ang II-treated H9C2 cells (Fig. 6 G − I). These findings suggest that SYT1 deficiency directly aggravates Ang II-induced cardiomyocyte apoptosis in vitro.

### Exacerbated myocardial p38 MAPK phosphorylation in Syt1^+/−^ mice in response to pressure overload in vivo

**Fig. 7:**
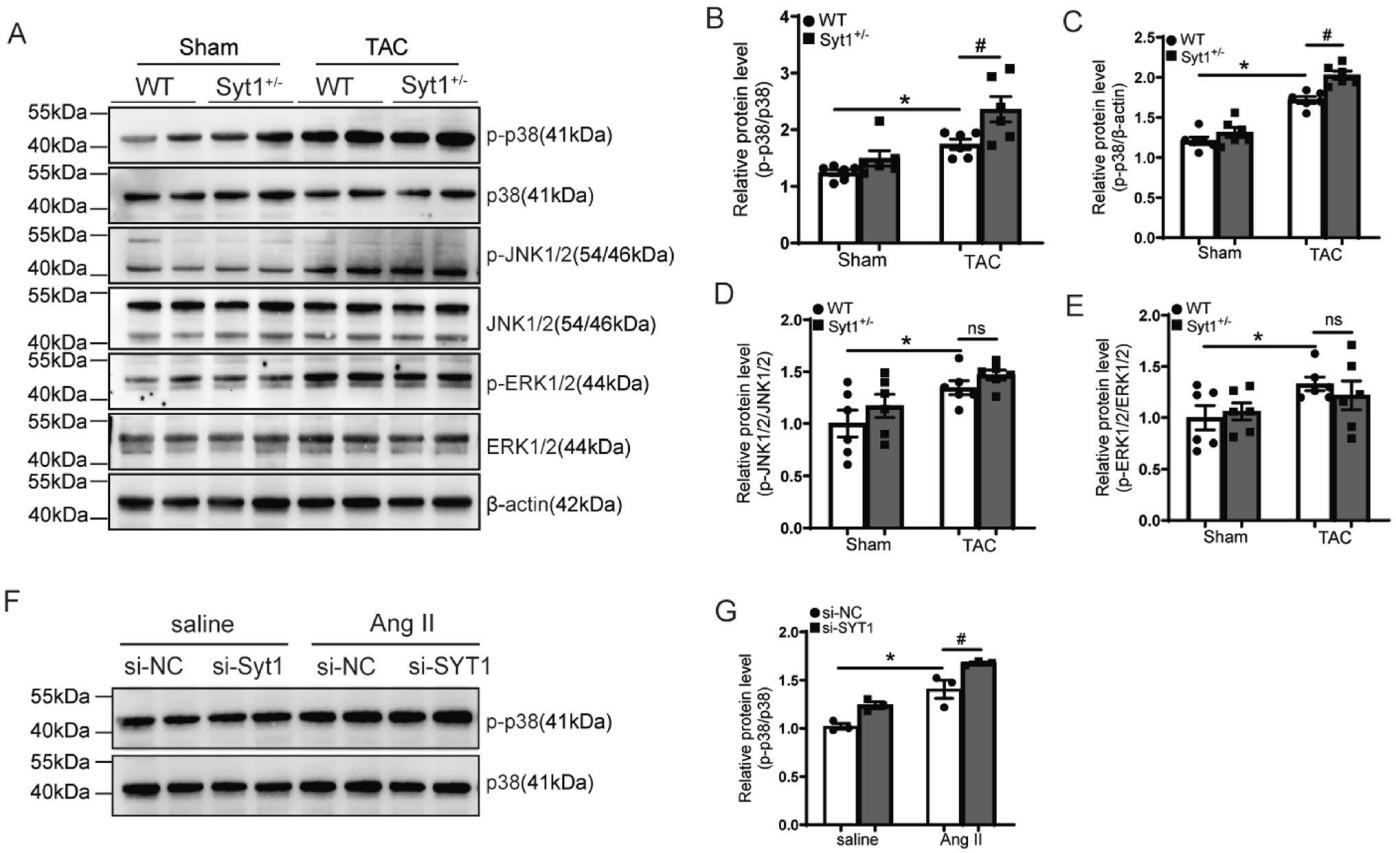
Altered phosphorylation of p38, JNK and ERK in *Syt1*^+*/−*^ mice with TAC. **A** representative western blots of p-p38, p38, p-JNK1/2, JNK1/2, p-ERK1/2 and ERK1/2 in the LV tissues of WT mice and *Syt1*^+/−^ mice eight weeks after TAC (*n* = 6).** B** through** E** quantitative analysis of results shown in (**A**) (*n* = 6). **F** and **G** representative western blots and quantification of the p38 phosphorylation in H9C2 cells infected with the indicated siRNAs and treated with saline or Ang II for 24 h (*n* = 3). Mean ± SEM. *P < 0.05. ^#^*P* < 0.05. *ns* not statistically significant

It is well known that the MAPK signaling pathway plays a vital role in the progression of cardiac hypertrophy, and SYT1 has been confirmed to regulate the MAPK signaling pathway in colorectal cancer [10]. However, whether SYT1 affects cardiac hypertrophy by regulating the MAPK signaling is unknown. The MAP kinase family consists of extracellular signal-regulated protein kinase (ERK), c-Jun NH2-terminal protein kinase (JNK), and p38 MAP kinase (p38). We therefore examined the phosphorylation and expressions levels of these representative proteins involved in the MAPK signaling pathway. Results showed that the phosphorylation level of myocardial p38 was significantly increased eight weeks after TAC in WT mice, and this effect was potentiated by SYT1 KO (Fig. 7A–C). Additionally, the phosphorylation of JNK and ERK were significantly increased in WT mice after TAC. However, there was no significant difference in the phosphorylation and expression levels of JNK1/2 and ERK1/2 between *Syt1*^+*/−*^ mice and WT mice after TAC (Fig. 7D, E). Consistent results were achieved in vitro. In Ang II-treated H9C2 cardiomyocytes, the phosphorylation level of p38 was increased, and silencing SYT1 (si-SYT1) exacerbated this increase of p38 phosphorylation (Fig. 7F, G). These results suggest that the pro-hypertrophic effect of SYT1 deficiency is mainly related to the p38 MAPK signaling sub-pathway, but not the ERK and JNK sub-pathways.

### Aggravation of cardiomyocyte hypertrophy and apoptosis by SYT1 deficiency via activating the p38 MAPK signaling in vitro

**Fig. 8:**
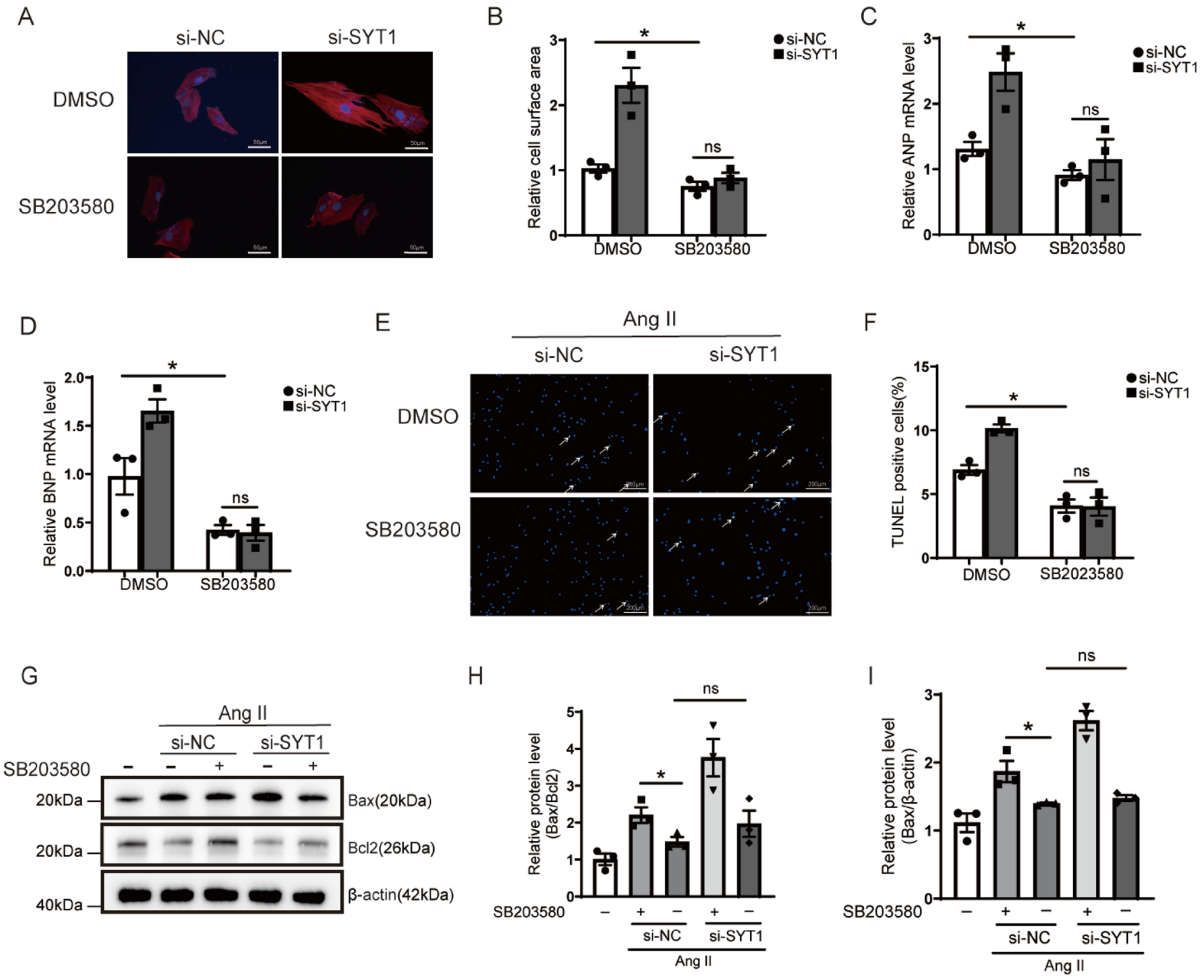
Enhancement of H9C2 cardiomyocyte hypertrophy and apoptosis and upregulation of p38 phosphorylation after SYT1 silencing. **A** and **B** immunofluorescent images of H9C2 cells and quantitative measurements of cell size in different treatment groups (*n* = 3). **C** and** D** RT-qPCR results of ANP and BNP levels in the H9C2 cells with different treatments (*n* = 3). **E** and **F** representative TUNEL staining images and quantification of TUNEL-positive cells in the indicated groups (*n* = 3). **G** through **I** representative western blots of Bax and Bcl2 levels and respective quantification in H9C2 cells with different treatments (*n* = 3). Mean ± SEM. *P < 0.05. *ns* not statistically significant

The above in vivo results showed that SYT1 KO enhanced the phosphorylation of p38 MAPK which contributed to TAC-induced cardiac hypertrophy, we next observed whether inhibition of p38 MAPK could rescue cardiomyocyte hypertrophy using SB203580, a specific inhibitor of p38 MAPK in the hypertrophic H9C2 cell model induced by Ang II. The results showed that SB203580 alleviated the enlargement of H9C2 cell surface area (Fig. 8A, B), and decreased the transcription levels of ANP and BNP (Fig. 8C, D). These results indicate that phosphorylation of p38 was indeed involved in SYT1 deficiency-associated cardiomyocyte hypertrophy. Furthermore, pretreatment with SB203580 rescued the enhanced apoptosis in *Syt1*-silencing H9C2 cells after Ang II stimulation, as indicated by reduction of TUNEL-positive cell proportion (Fig. 8E, F). There was no significant difference in the Bax and Bcl-2 protein levels between the Ang II + si-NC + SB203580 cell group and the Ang II + si-SYT1 + SB203580 group (Fig. 8G − I). Collectively, these findings indicate that SYT1 deficiency aggravates cardiomyocyte hypertrophy and apoptosis at least partially by activating the p38 MAPK signaling pathway.

## Discussion

Cardiac hypertrophy is an independent risk factor for heart failure and sudden death. Many detrimental stimuli can induce cardiac hypertrophy, such as hypertension, myocardial injury, neurohumoral overactivation, and genetic mutation [13]. In basic studies, pressure overload is the most commonly used stimulus to create animal models of cardiac hypertrophy, as it significantly increases the afterload of the heart and leads to reduction in chamber dimension and increment in wall thickness [2, 14]. In the present study, TAC for eight weeks induced cardiac hypertrophy with deceased cardiac performance and other cardiac remodeling features including fibrosis and cardiomyocytes apoptosis. Cardiomyocyte hypertrophy and apoptosis may spatiotemporally present in the hypertrophic heart. TAC also elevated the myocardial and serum Ang II levels in mice, suggesting a role of Ang II in TAC-induced cardiac hypertrophy. We investigated the role of SYT1 in the development of TAC-induced cardiac hypertrophy in mice in vivo and Ang II-induced cardiomyocyte hypertrophy in H9C2 cells in vitro, and found that *Syt1* KO or silencing exacerbated cardiac hypertrophy, dysfunction, fibrosis and cardiomyocyte apoptosis, strongly suggesting that SYT1 is a protective factor of cardiac hypertrophy. Mechanistic experiments showed that the aggravation of TAC-induced cardiac hypertrophy and dysfunction by SYT1 KO or silencing is mediated by activated p38 MAPK signaling pathway, the major pathway that induces cardiac hypertrophy.

SYT1 is traditionally considered a Ca^2+^ sensor in the process of neurotransmitter release and cellular vesicle exocytosis. For example, SYT1 plays an important role in evoked, synchronous neurotransmitter release in neurons [15]. Mutations of SYT1 in patients have been found associated with mental abnormalities. Baker et al. and Bradberry et al. reported the clinical phenotypes in 11 individuals with *Syt1* mutation and confirmed that *Syt1* mutation is associated with a recurrent neurodevelopmental disorder [16, 17]. In recent years, SYT1 has been found expressed in a variety of non-neuronal tissues or cells and has biological functions. For example, SYT1 is required for spindle stability [18] and regulates cortical granule exocytosis during mouse oocyte activation [19]. SYT1 is involved in hepatic management of lipids [20]. However, rare information is available about the expression and function of SYT1 in the myocardium and cardiomyocyte. Peters et al. reported the expression of SYT1 in atrial cardiac myocytes in 2006, but they did not investigate its function in the atrium [21]. Other members of the SYT family have been reported to play a role in the heart. Our group recently reported that synaptotagmin-7 was expressed in mice ventricular myocytes and mediated cardiac hypertrophy by targeting autophagy [22]. The intronic variant rs11041321 of the *Syt9* gene was associated with an increased risk of developing congenital cardiac septal defects (CCSDs) in Han Chinese populations [23]. Repression of *Syt17* transcription resolves established cardiomyopathy [24]. However, the role of SYT1 in the heart remains uncharacterized and whether SYT1 participates in the cardiac remodeling is unclear. In the present study, we generated a *Syt1*^+*/−*^ mice strain and used it to assess the potential role of SYT1 in cardiac hypertrophy. We discovered that SYT1 was expressed in mice ventricles and H9C2 cardiomyocytes; SYT1 KO exacerbated TAC-induced cardiac hypertrophy in mice; SYT1 silencing also exacerbated Ang II-induced cardiomyocyte hypertrophy and apoptosis. To our knowledge, this is the first report to demonstrate the role of SYT1 in cardiac hypertrophy at the integrative and cellular levels.

We observed here that SYT1 deficiency exacerbated cardiac hypertrophy in vivo and cardiomyocyte hypertrophy in vitro, suggesting that SYT1 has a function of repressing pressure overload-induced cardiac hypertrophy. Notably, we found that heterozygous SYT1 KO alone could not induce cardiac hypertrophy. This phenomenon is often seen in some alternative studies, in which knockout (KO) or knockdown (KD) of certain genes could not cause significant pathological phenotypes, unless the animals or cells are challenged by additional stressors. For example, cardiac-specific deletion of USP28 aggravates diabetic heart failure and mitochondrial dysfunction only when the mice were challenged with high-fat diet/streptozotocin, indicating that the knockout phenotype was stress-dependent [25]. Similarly, deletion of MCU did not exhibit any overt baseline phenotype but exacerbated cardiac hypertrophy and cardiomyocyte death in mice subjected to continuous isoprenaline infusion, again demonstrating the importance of stress in revealing knockout phenotypes [26]. The above studies suggest that KO or KD of a certain gene may not be enough to induce abnormality, but increase the susceptibility to a stressor. The reason behind this phenomenon may likely be that certain genes may have redundant functions and compensatory mechanisms, which may mitigate the effects of gene loss under normal conditions. However, when additional stressors are introduced, these compensatory mechanisms may be overwhelmed, revealing the underlying vulnerabilities.

The pathology of cardiac hypertrophy is complex and is associated with excessive cardiomyocyte apoptosis, except for cardiomyocyte hypertrophy and cardiac fibrosis. Apoptosis of cardiomyocytes has been reported to be a main pathological process leading to cardiac hypertrophy [27]. Due to limited capability of cardiac regeneration, cardiomyocyte death induced by apoptosis directly contribute to cardiac dysfunction or even heart failure [28]. Previous researches have shown that SYT1 is involved in apoptosis of numerous cell types. SYT1 exerts a neuroprotective effect by suppressing neuronal apoptosis. Inhibition of lung SYT1 expression enhances lung injury and apoptosis [29]. Here we showed that SYT1 KO aggravated cardiomyocyte apoptosis in the situations of pressure overload or Ang II stimulation, as evidenced by the TUNEL staining and the Bax/Bcl-2 ratio.

P38, a stress activated Ser/Thr kinase, can be phosphorylated and thus activated under stress and inflammatory stimuli [30, 31]. The function of p38 has been studied in depth in relation to cardiac hypertrophy. Numerous reports have demonstrated that the p38 MAPK pathway is activated in cardiomyocytes exposed to hypertrophic stimuli [32], and cardiomyocyte hypertrophy is attenuated by p38 MAPK inhibition using pharmacological inhibitors SB203580. Ang II induces phosphorylation and activation of p38 kinases in the heart, and cardiac knockout of p38 prevents cardiac hypertrophy in vivo [33]. In addition, acute activation of endogenous p38 MAPK in adult heart results in cardiac hypertrophy and contractile dysfunction [34]. Therefore, p38 MAPK activation is closely related to cardiac hypertrophy. However, a connection between p38 MAPK and SYT1 has not been established. We showed here that the phosphorylated p38 (p-p38) level was significantly increased in the myocardium of TAC mice and in Ang II-stimulated H9C2 cardiomyocytes, while SYT1 KO or silencing exacerbated these effects, suggesting that SYT1 can adversely regulate cardiac hypertrophy mainly via affecting the phosphorylation of p38. Inhibition of p38 by SB203580 in H9C2 cardiomyocytes rescued the SYT1 silencing-induced cell hypertrophy and apoptosis, conversely proved the key role of p38 for SYT1 to regulate cardiac hypertrophy and cardiomyocyte apoptosis. However, our data demonstrate no significant changes for other MAPK members (such as ERK and JNK) in SYT1 KO mice. Despite their similar structural organization, the conventional MAPKs, including p38, ERK and JNK, have distinct regulation and functions. For p38, MKK3 and MKK6 are thought to be the major protein kinases responsible for activation, which are distinguished from other MAPK members. In addition, biological functions of the MAPKs differed. Studies have reported that p38 is the MAPKs most associated with apoptosis [35]. Consistent with these reports, our results showed that SYT1 deficiency aggravates cardiomyocyte apoptosis mainly by activating p38, no other MAPKs, providing further evidence for pro-apoptosis functions of p38. However, the mechanisms of how SYT1 function through p38 MAPK pathway need to be further studied.

### Limitations

The first limitation of the study is that we used Ang II but not mechanical stretch to induce cardiomyocyte hypertrophy. Mechanical stretch is more appropriate than Ang II in simulating pressure overload, although Ang II was also frequently used to induce cardiomyocyte hypertrophy. The second limitation is that we did not examine the effect of angiotensin receptor blocker in TAC-induced myocardial SYT1 elevation at the integrative level, although we found that TAC induced elevations of myocardial and serum Ang II levels in mice, and Ang II increased SYT1 expression and losartan repressed this effect of Ang II in H9C2 cells. These results suggest the involvement of angiotensin signaling in pressure overload-induced cardiac hypertrophy.

In summary, the present study identified a previously unrecognized biological function of SYT1 in cardiac hypertrophy. SYT1 deficiency aggravates pressure overload-induced cardiac hypertrophy, fibrosis, dysfunction, and cardiomyocyte apoptosis mainly by activating the p38 MAPK pathway. The study suggests a protective role of SYT1 in cardiac hypertrophy and provides promising therapeutic targets for cardiac hypertrophy.

## Supplementary Information

Below is the link to the electronic supplementary material.

Supplementary file1 (DOCX 2501 KB)
