# Supplementary material for: Deficiency of synaptotagmin-1 aggravates pressure overload-induced cardiac hypertrophy and dysfunction via the p38 MAPK signaling pathway in mice
Source: Hum Cell. 2025 Apr 25;38(3):96. doi: 10.1007/s13577-025-01220-z (PMC12031904; doi:10.1007/s13577-025-01220-z)

**Deficiency of synaptotagmin-1 aggravates pressure overload-induced cardiac hypertrophy and dysfunction via the p38 MAPK signaling pathway** **in mice**

Jing Shen^1,^**^#^**, Junqiu Miao^1,#^, Lifei Wu^1^, Deping Wang^1^, Guang Li^2,^**^*^**, Haixiong Wang^3,*^, and Jimin Cao^1,^**^*^**

^1^ Key Laboratory of Cellular Physiology at Shanxi Medical University, Ministry of Education, and the Department of Physiology, Shanxi Medical University, Taiyuan 030001, China

^2^ Key Laboratory of Medical Electrophysiology at Southwest Medical University, Ministry of Education, and the Institute of Cardiovascular Research, Southwest Medical University, Luzhou 646099, China

^3^ Department of Cardiology, Shanxi Cardiovascular Hospital, Taiyuan 030000, China

^#^ These authors contributed equally to the work.

**^*^** Corresponding author. Email: caojimin@sxmu.edu.cn (J. C.) (lead contact); Email: [liguang@swmu.edu.cn](mailto:liguang@swmu.edu.cn) (G. L.); cz1976whx@126.com (H.W.)


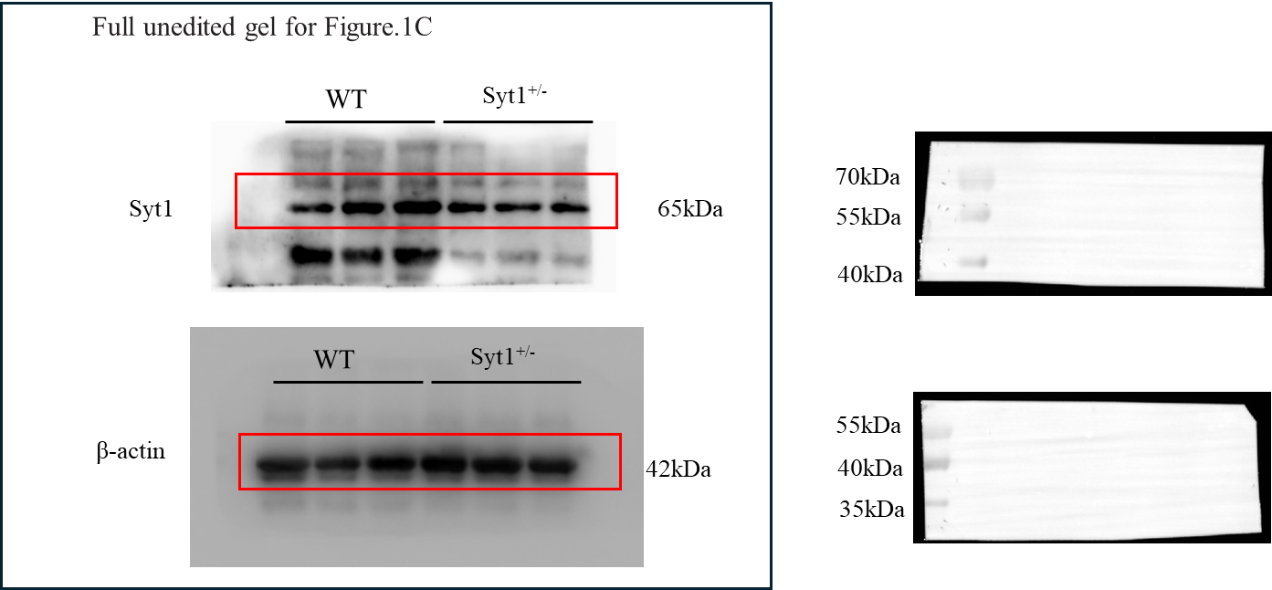


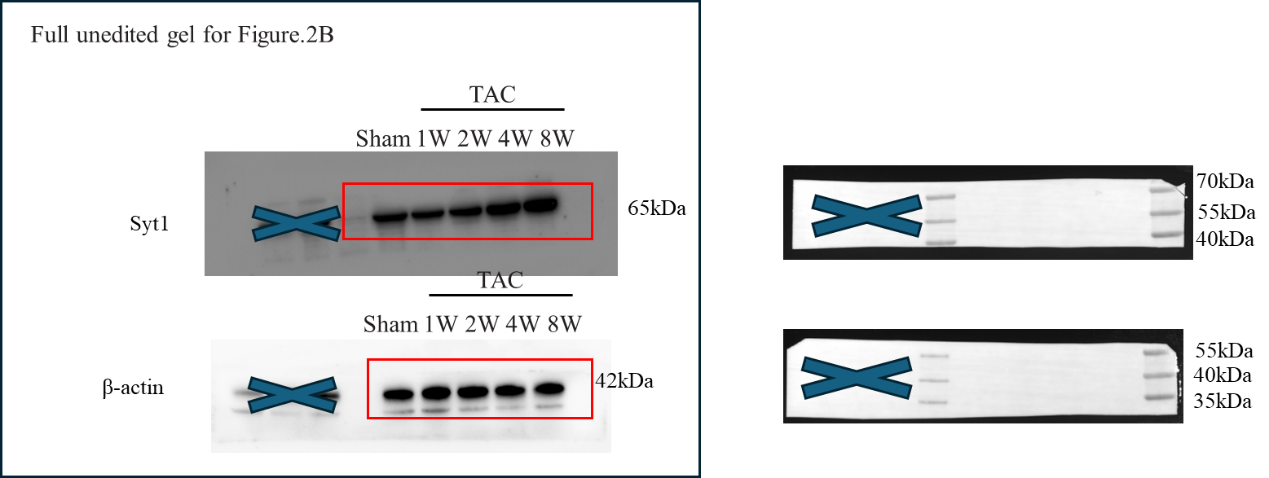


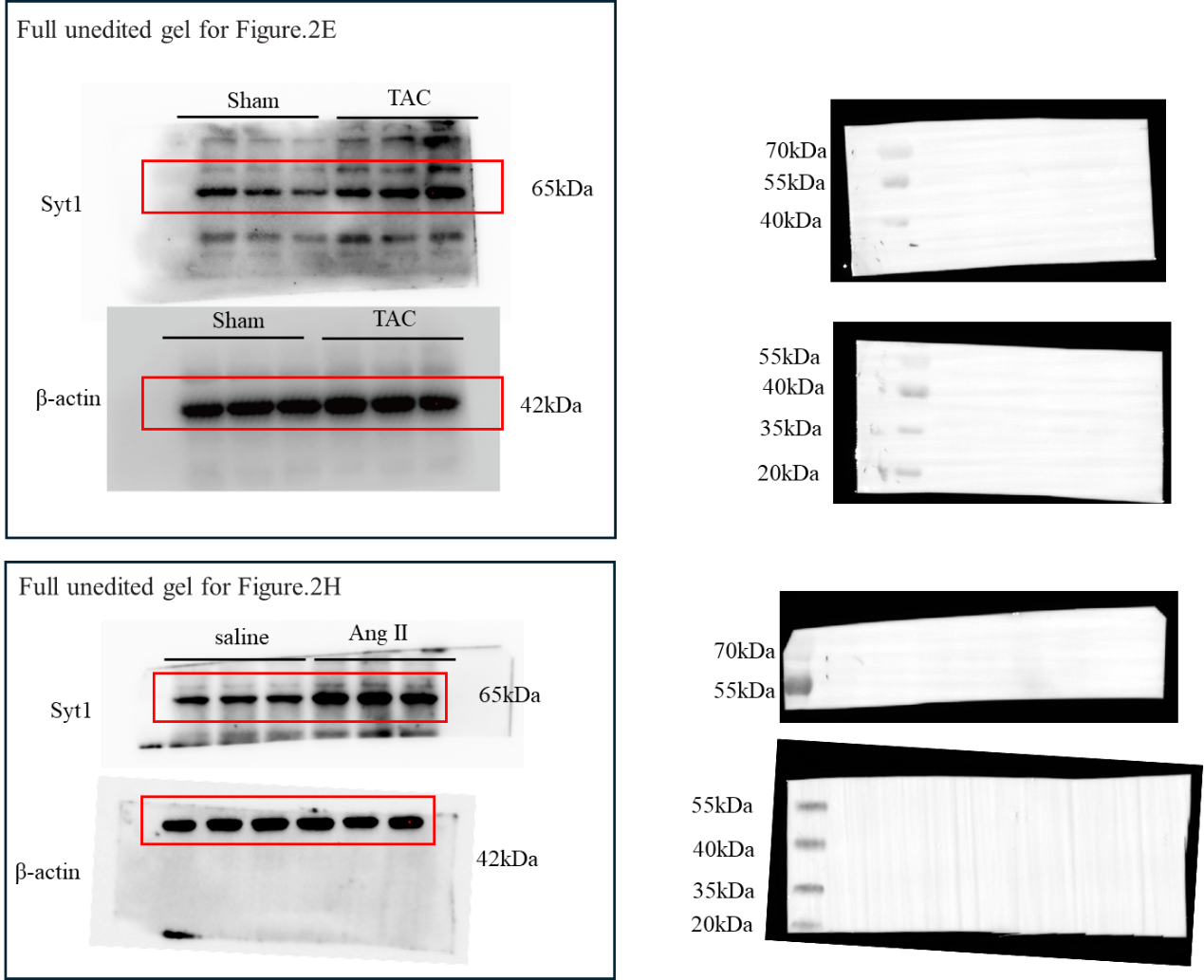


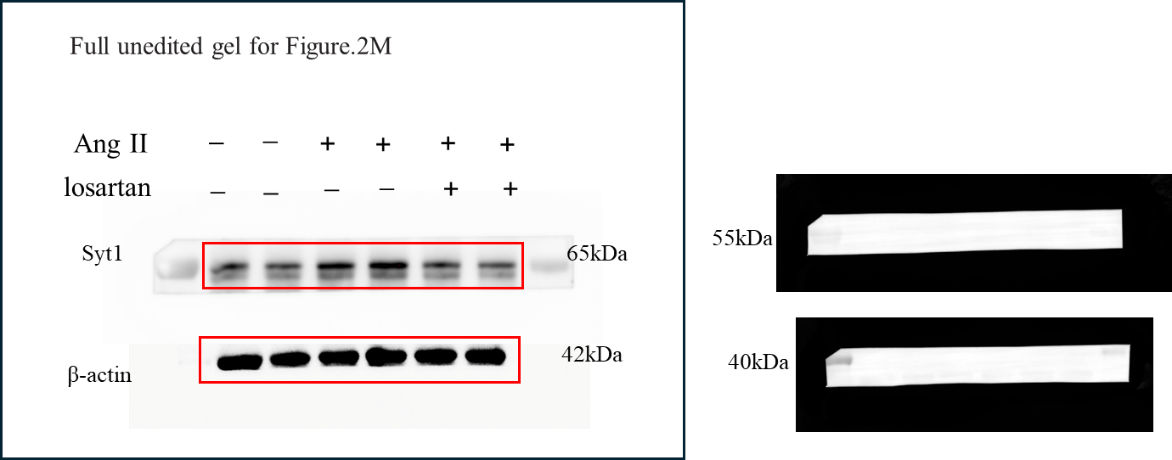


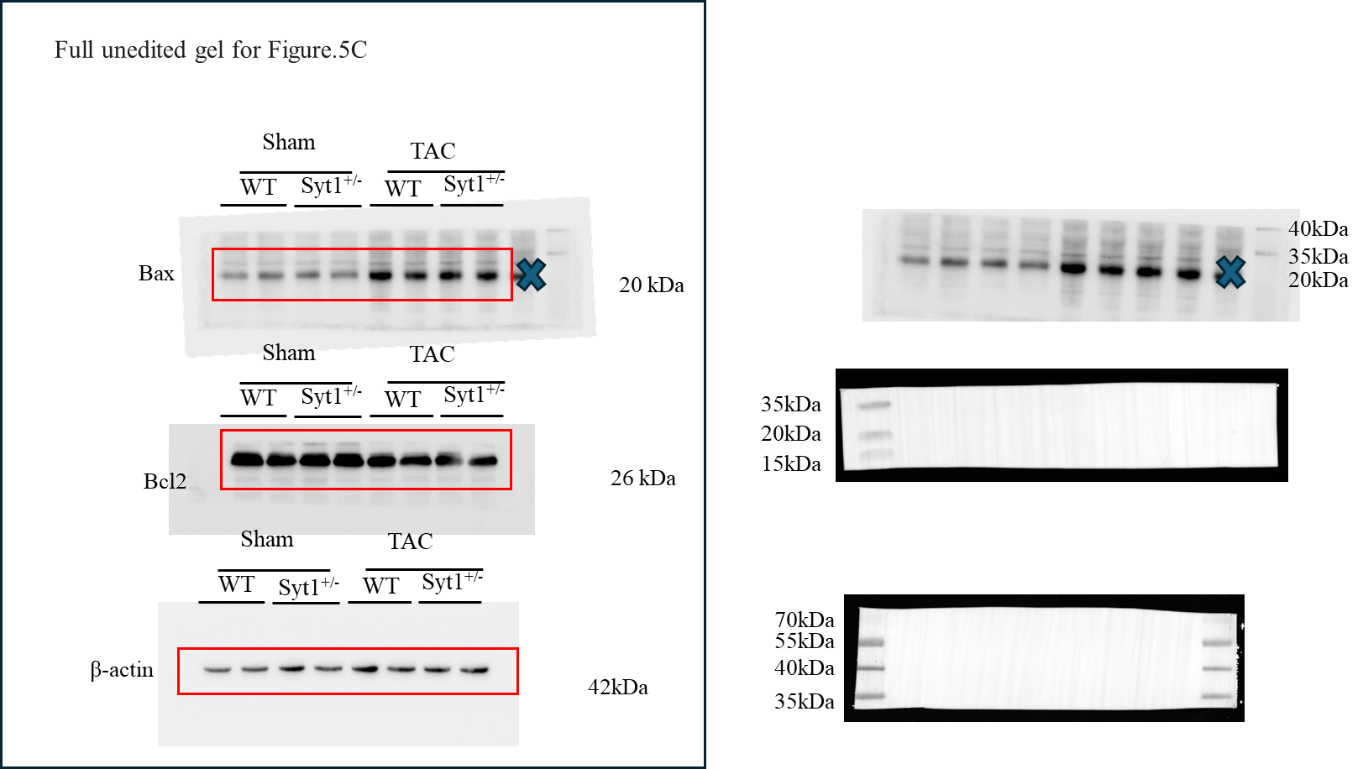


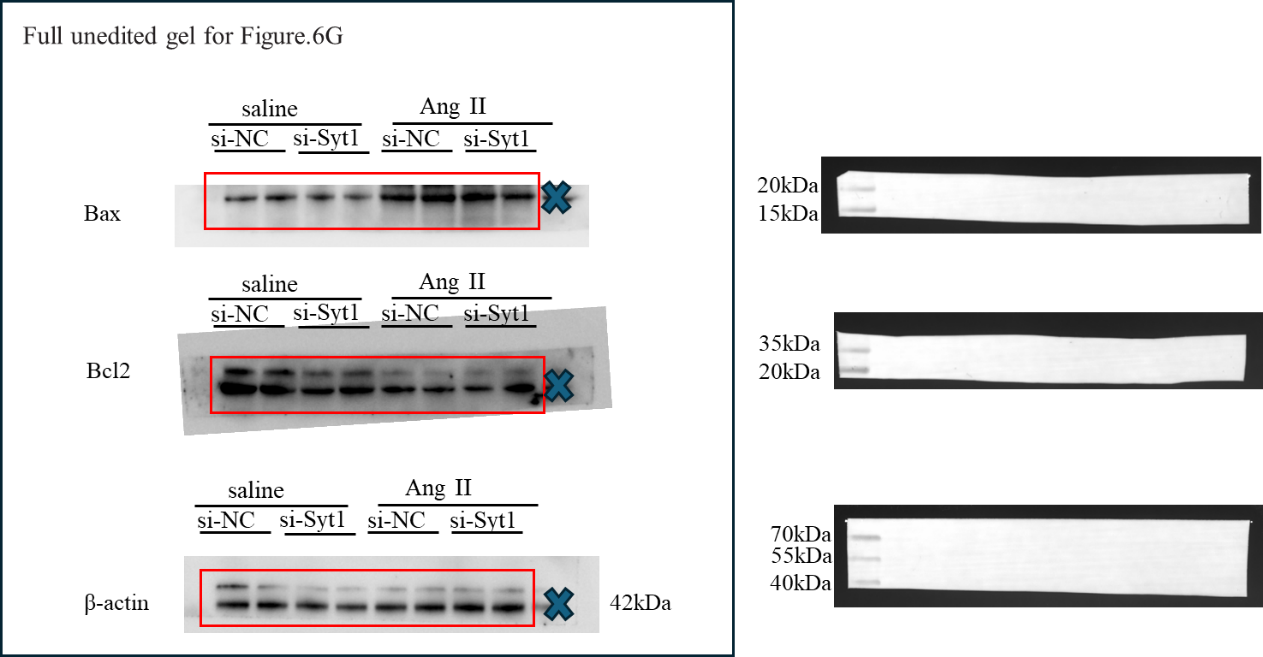


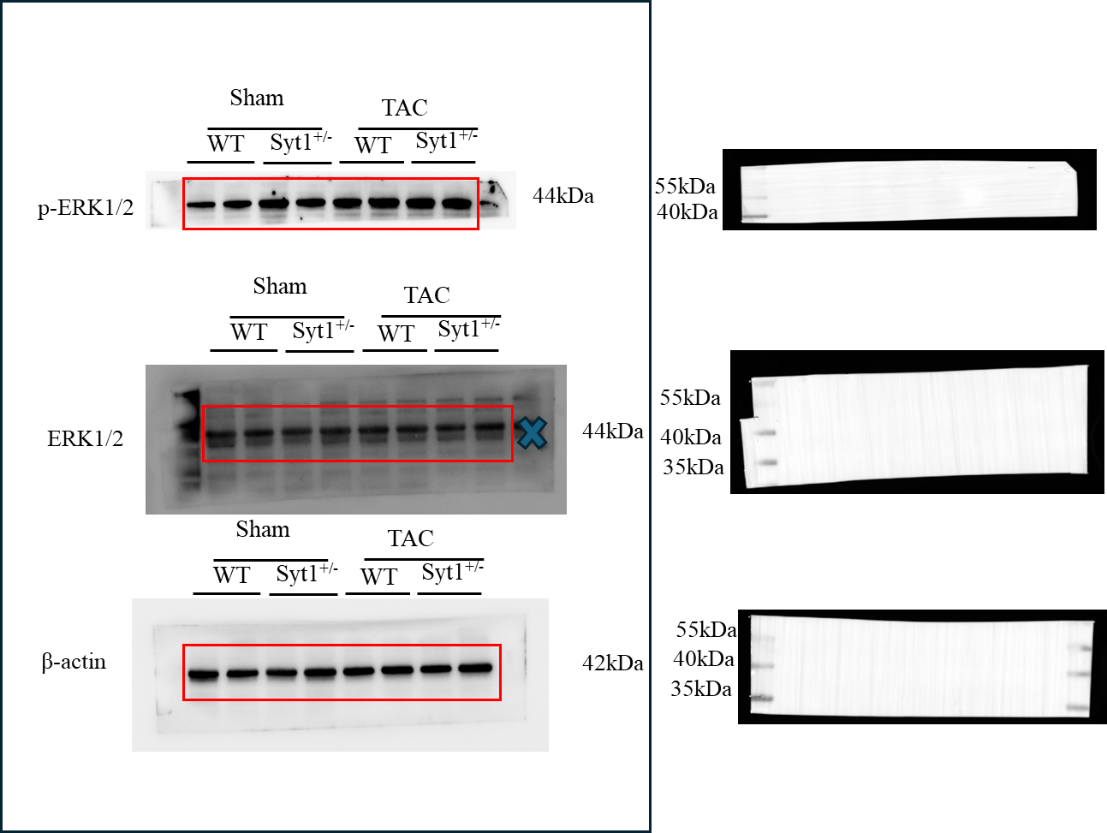

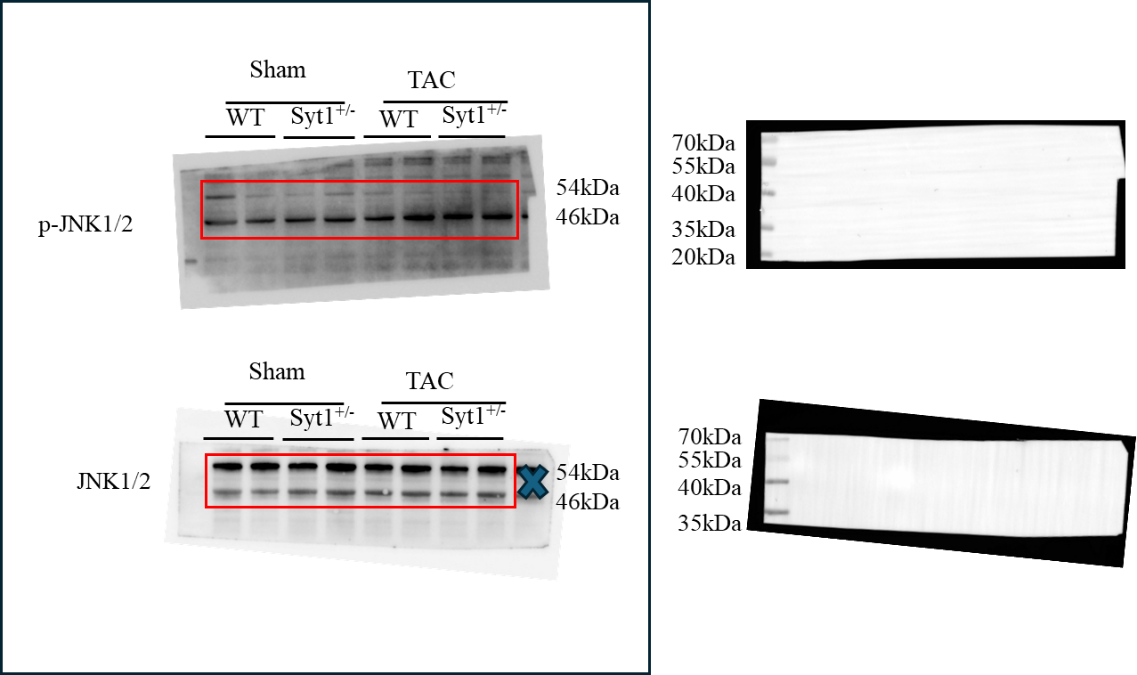

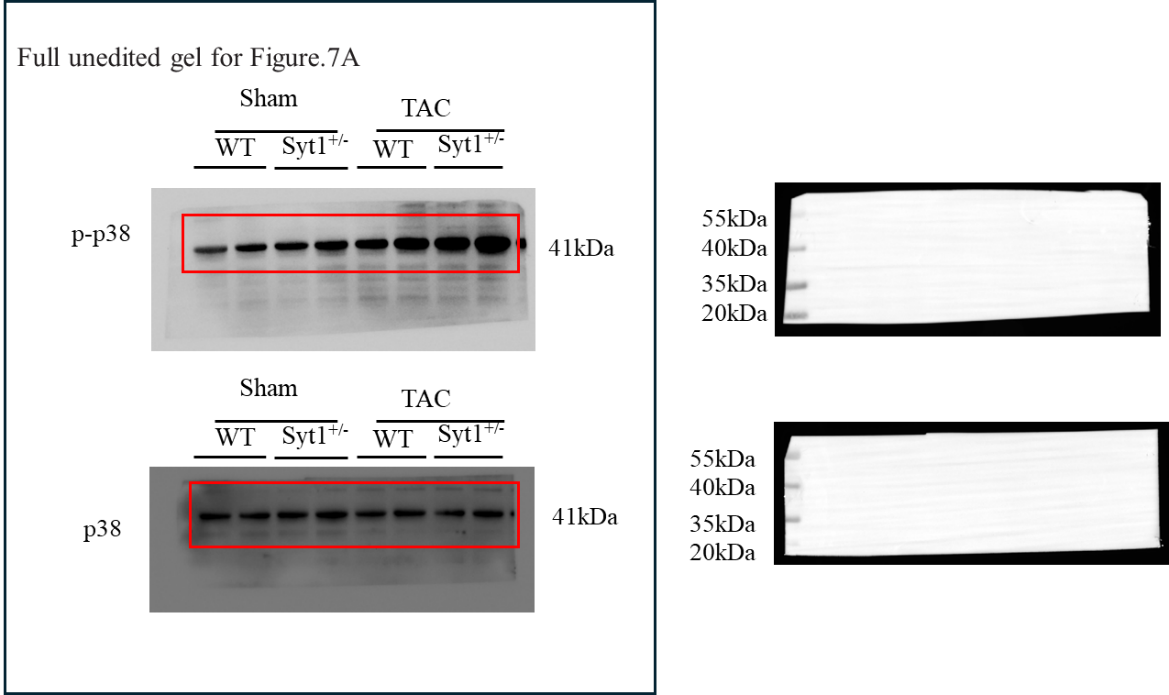


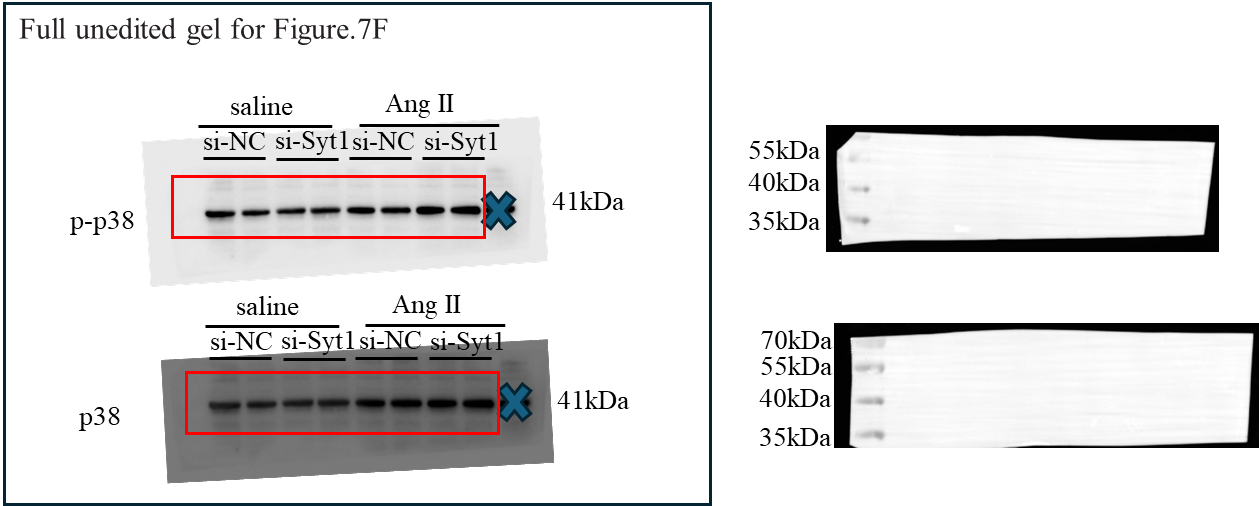


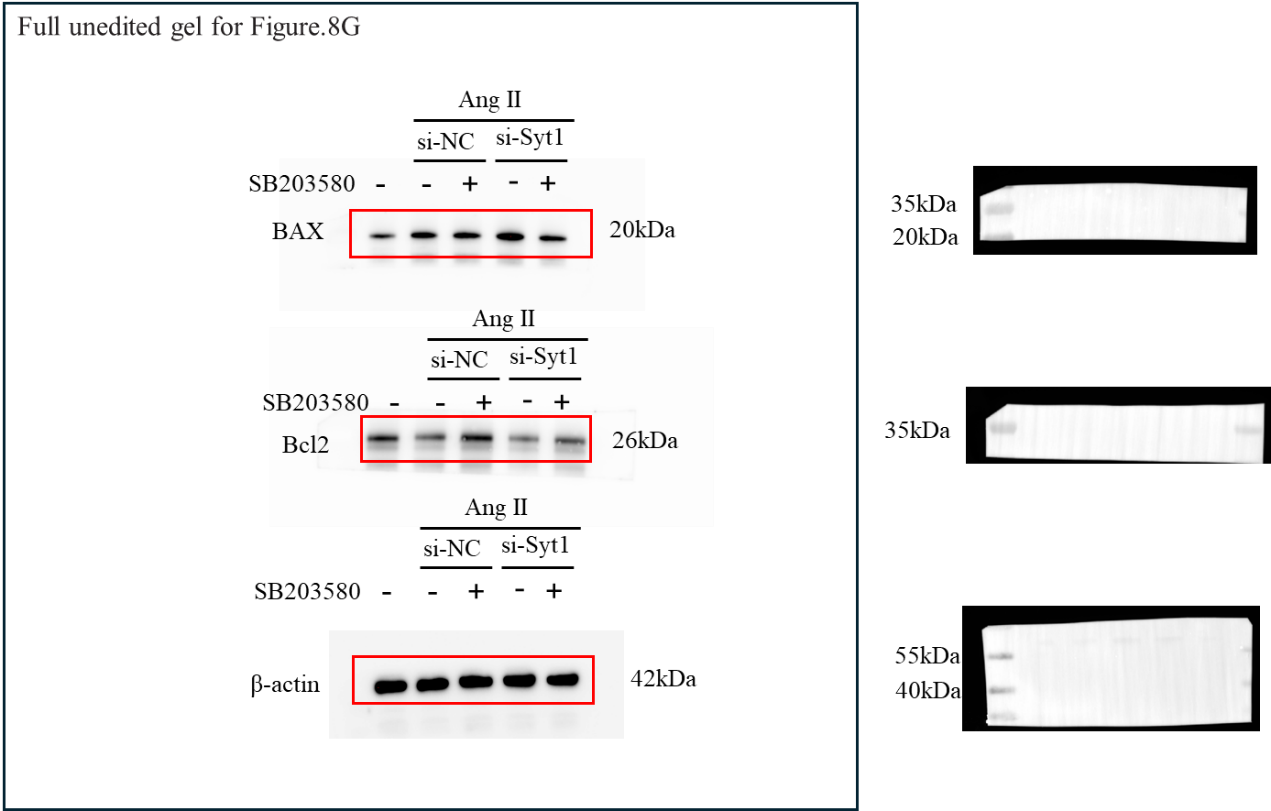

Supplement: Supplementary file 1 — Supplementary file1 (DOCX 2501 KB) [file 13577_2025_1220_MOESM1_ESM.docx]
